# Sensitivity to Auditory Velocity Contrast

**DOI:** 10.1038/srep27725

**Published:** 2016-06-13

**Authors:** Shannon M. Locke, Johahn Leung, Simon Carlile

**Affiliations:** 1 School of Medical Sciences, University of Sydney, NSW 2006 Australia; 2 Department of Psychology, New York University, 6 Washington Place, New York, NY 10003, USA; 3 Starkey Hearing Research Center, 2110 Shattuck st#408, Berkeley, CA 94704 USA

## Abstract

A natural auditory scene often contains sound moving at varying velocities. Using a velocity contrast paradigm, we compared sensitivity to velocity changes between continuous and discontinuous trajectories. Subjects compared the velocities of two stimulus intervals that moved along a single trajectory, with and without a 1 second inter stimulus interval (ISI). We found thresholds were threefold larger for velocity increases in the instantaneous velocity change condition, as compared to instantaneous velocity decreases or thresholds for the delayed velocity transition condition. This result cannot be explained by the current static “snapshot” model of auditory motion perception and suggest a continuous process where the percept of velocity is influenced by previous history of stimulation.

To the brain, most sounds are in motion. This is because both source-motion in the external world and self-motion from head movements give rise to identical dynamic acoustical cues. Numerous studies have examined sensitivity to the velocity of a moving sound^1^,^2^,^3^,^4^,^5^,^6^,^7^,^8^, with the broadly accepted finding that a 20–70% difference in velocity is necessary for discrimination^8^. The common psychophysical approach is to ask subjects to compare two constant velocity sounds, with distinct start and end points that are presented sequentially. Given the lack of physiological evidence for neurons that specifically encode speed (see ref. 9 for a review) and the insensitivity to velocity^6^, it is commonly accepted that velocity is estimated using distance cues.

Grantham^10^ proposed these distance cues were independent static snapshots of the sound’s location at the start and end points in what has been termed the two-snapshot model. However, in a natural auditory scene, speed often varies over time, and such a post-hoc inference strategy would be insensitive to velocity changes. Perrott, Constantino, and Ball^11^ motivated extensions of Grantham’s model to include snapshots during motion (i.e. a multi-snapshot model), by showing subjects could discriminate between randomly generated accelerations and decelerations. Their study, however, did not examine the magnitude of change in velocity necessary for discrimination. Here, we measured sensitivity to velocity changes during motion to examine how the velocity of a moving sound is perceived on a moment-to-moment basis. Importantly, listeners were unable to use the start and endpoints to make their judgments as was possible in previous velocity discrimination tasks.

We designed a velocity contrast task that was presented in virtual auditory space^12^. Unlike the standard velocity discrimination paradigm where the two stimulus intervals have the same spatial arc, we aligned the intervals along one common trajectory. That is, a step change in velocity was presented during a single sound movement, instead of two temporally and spatially distinct moving sounds with constant velocities. This allowed us to examine not only the sensitivity to the magnitude of change but also compare the direction of change (increase or decrease). Two temporal profiles were examined: the sound intervals were separated by a 1 second silent gap in the discontinuous condition, or without a gap in the continuous condition. The discontinuous condition is analogous to previous velocity discrimination experiments in that the start and end point cues were informative. Therefore, we expected subjects to have similar levels of sensitivity in the discontinuous condition as reported for the standard velocity discrimination task. In the continuous condition, we predicted the absence of start and end point cues would dramatically impair velocity discrimination.

Our results showed a significantly impaired performance in the continuous condition, but only for increases in velocity: the just noticeable differences (JNDs) were more than three times larger compared to the comparable discontinuous condition and five times larger than those reported previously^8^. This finding is not consistent with the currently received view of auditory motion perception and suggests a more continuous process where the instantaneous percept of velocity is significantly influenced by the previous history of stimulation.

## Materials and Methods

### Participants

Five listeners (two female, 20–39 years old) were recruited. Two listeners were authors while the rest were naive to the details of the study. All participants reported normal hearing and gave written informed consent. The methods were carried out in accordance with the approved guidelines of the University of Sydney Ethics Committee. All experimental protocols were approved by the University of Sydney Ethics Committee.

### Experimental Setup

Participants were seated in a darkened sound-attenuating chamber offering 40 dB of attenuation from 300 Hz upwards. They faced a 15-inch computer monitor, which displayed a grey fixation cross on a black background. Stimuli were generated using MATLAB (Mathworks^TM^) and presented over Beyerdynamic DT990 headphones, via a RME Fireface 400 audio interface. Sounds were presented at a comfortable listening level. Responses were made using a standard computer keyboard.

### Psychophysical Task

**Figure 1:**
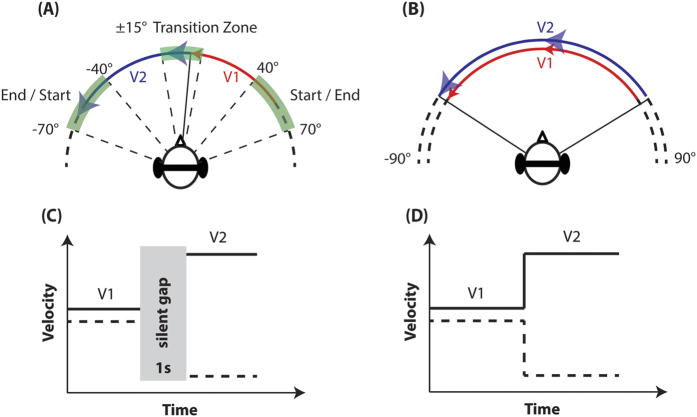
(**A**) Diagram showing the velocity contrast task design. Subject’s head remained stationary while the stimuli moved along the frontal audio-visual horizon, using virtual auditory space. The start and end points were ±40–70°, depending on velocity, with a ±15° transition zone in the middle. There were two stimulus intervals along the movement trajectory that had different velocities, V1 and V2 (marked red and blue). In an increase condition, V2 > V1, and V2 < V1 in a decrease condition. (**B**) An example of a velocity discrimination task design that is used in previous studies. Unlike a velocity contrast task (**A**), the two stimulus intervals in (**B**) travel along similar trajectories (marked red and blue), separated in time by an inter stimulus interval (ISI). (**C**) Velocity profile of the discontinuous condition. The two intervals (V1 and V2) are separated in time by a 1 second gap of silence; this is identical to the ISI used in Carlile and Best^8^. (**D**) Velocity profile of the continuous condition. The two intervals (V1 and V2) are continuous in time (and space).

All stimuli were presented in the frontal audio-visual horizon, with the direction of motion alternating from trial-to-trial to prevent adaptation. Start and endpoints were randomly selected from ±40–70°, and the velocity transition point varied between ±15° around the midline (see Fig. 1). The initial velocity interval is denoted as V_1_ and the final velocity interval as V_2_. The velocity transition was either discontinuous or continuous (Fig. 1C,D respectively). In the continuous transition condition, the V_1_ and V_2_ intervals were temporally contiguous, and the change between the velocities was immediate. In the discontinuous transition condition, the intervals were separated by 1 second of silence, identical to the previous velocity study conducted by our lab^8^. We expected this to provide a clear velocity percept for each interval to act as a baseline comparison.

Listeners completed a yes-no task with a 2 × 2 × 2 randomized block design. The variables examined were: 1) reference velocities: 30°/s versus 60°/s, 2) direction of velocity change: increase versus decrease, 3) transition type: continuous versus discontinuous. Each condition was presented as a separate block, the order randomized for each listener. Listeners were asked to report if they perceived the target velocity change, which was either an increase or decrease depending on the block. The two reference velocities, examined were 30 and 60°/s. Ten test velocities were examined for each reference velocity, half faster and half slower (see Table 1).

If the subject did not reach 75% correct or the sampling was inadequate to characterize the threshold, an additional block of that condition was completed at the end of the experiment. In order to compute the sensitivity measure d-prime, catch trials were randomly interspersed (see supplementary material). A block contained 10 repeats of each of the 5 target trial combinations (50 in total) and 15 catch trials. Each block took an average 45 minutes and the whole experiment averaged 360 minutes, Subjects were provided ample rest breaks between blocks.

### Data Analysis

Performance was analyzed using the d-prime measure. The mean error rate across the catch trials was used as the false alarm rate for each threshold calculation. The d-prime values were fitted with an isotonic regression and the threshold was the stimulus intensity with a d-prime of 1.35, corresponding to approximately 75% correct rate^13^. Importantly, we were able to compare the sensitivity between increases versus decreases in velocity, by separately calculating an upper and lower threshold for each reference velocity tested. As there were no significant differences between leftward and rightward motion the results were combined. Analysis was performed on the group mean data.

### Stimulus Generation

All moving sounds were generated in Virtual Auditory Space (VAS) using individualized Head Related Transfer Functions (HRTFs)^12^. This provided the flexibility in stimulus placement and velocity control that was necessary for the task. Sounds in VAS are well externalized as they contain location-dependent spectral filtering cues in addition to interaural time and level differences. Prior to the experiments outlined here, each participant had their HRTFs recorded using a procedure detailed in^14^. Briefly, this involved playing an exponential sine sweep burst (200 Hz–20 kHz) from a moveable Audience A3 speaker at a distance of 1.1 meters. This stimulus was sequentially presented at each degree between −90° to 90° in the horizontal plane. Two small microphones, seated in the participant’s ear canals, recorded the incidence wave of the sound arriving at the left and right ears. All recordings were conducted in a 64 m^3^ anechoic chamber that absorbed 99% of incident sound energy above 300 Hz. During post processing, the location independent components such as the headphone to eardrum transfer functions were removed, leaving only the directionally dependent information (see ref. 15).

To generate a virtual sound at the location θ, the intended auditory stimulus was convolved with the left and right ear HRTFs for θ. In both experiments, the stimulus was 300 Hz–16 kHz broadband noise, with a 48 kHz sampling rate. A 5 ms cosine ramp and amplitude modulation were also applied. When the filtered sounds are played through the corresponding left and right headphone channels, this gives the percept of a static sound at θ. Motion is generated by sequentially playing static sounds in 1° increments corresponding to the trajectory of the stimulus. This evokes the percept of smooth, continuous motion. The direction of motion is determined by the temporal ordering of the static sounds, and the speed by the duration the sound is played at each location.

## Results

**Figure 2:**
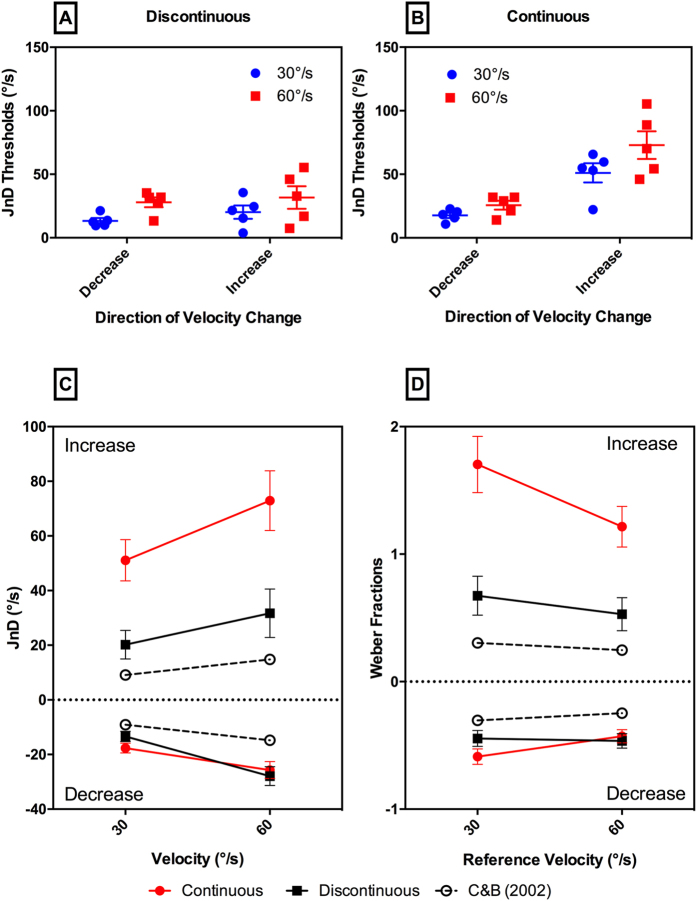
(**A**) Just noticeable difference (JND) thresholds for the Discontinuous condition, comparing between reference velocities (blue = 30°/s, red = 60°/s) and velocity direction (Decrease = left group, Increase = right group). Individual results are shown. (**B**) Similar to (**A**) but for JND Thresholds for the Continuous condition. (**C**) Average JND thresholds versus reference velocity, also shown are thresholds for Carlile and Best^8^. (**D**) Weber fractions versus reference velocity.

The results of this experiment are presented in Fig. 2. Plotted in Fig. 2A,B are the individual and mean JNDs. Figure 2C,D show the group summaries of JNDs and Weber fractions respectively, alongside the results from Carlile and Best^8^. In general, the thresholds reported here are larger than those found in this previous study. The largest mean threshold, 72.9 ± 10.9°/s (SEM), was observed in the continuous increase condition for the reference velocity of 60°/s. For comparison, Carlile and Best found a threshold of 14.8°/s for this reference velocity.

Data was analyzed with 2 × 2 × 2 repeated measures ANOVAs with the factors: transition type (continuous, discontinuous), direction of velocity change (decrease, increase), and reference velocity (30°/s, 60°/s). This analysis was performed with both raw thresholds and Weber fractions as dependent variables. A main effect of reference velocity was found in the raw thresholds ANOVA (F(1,4) = 30.7, p < 0.01), but was not significant in the Weber fractions ANOVA. Carlile and Best^8^ also found no significant differences between reference velocities when analyzed in terms of Weber fractions. Both ANOVAs, however, showed a significant interaction effect between transition type and velocity change direction: F(1,4) = 9.0, p < 0.05, for the raw thresholds analysis, and F(1,4) = 11.8, p < 0.05, for the Weber fraction analysis. For simplicity, the post hoc statistical tests were performed only on the results expressed as Weber fractions.

The significant interaction between transition type and change direction was analyzed using pair t-tests with Holm adjusted p-values. Weber fractions for continuous increases were found to be significantly greater than continuous decreases, t(4) = 8.7, p < 0.01; discontinuous increases, t(4) = 6.9, p < 0.01; and discontinuous decreases, t(4) = 8.3, p < 0.01. The continuous increase conditions had a mean Weber fraction of 1.50 ± 0.53, whereas the Weber fractions of the other conditions were 0.51 ± 0.16, 0.60 ± 0.35, and 0.46 ± 0.14 respectively. This corresponds to an approximate threefold increase in thresholds for the continuous increase conditions.

## Discussion

This study showed that listeners could discriminate changes in velocity that occurred during motion - if given a step change of sufficient magnitude. This corroborates the main conclusion from Perrott *et al*.^11^ that the two-snapshot model is insufficient to describe how humans perceive the velocity of moving sounds on a moment-to-moment basis because it would render them unable to perceive such velocity changes. The role of start and end points, however, can still be an important cue in velocity perception. The discontinuous transition condition (i.e. control condition) in our experiment allowed subjects to use this cue. The pattern of results on the discontinuous condition matches those previously reported in the literature. As in Carlile and Best^8^, there were no significant differences between velocity increases and decreases and thresholds increased with reference velocity.

Surprisingly, removing start and end point cues did not impair performance when the velocity decreased. Thresholds for the continuous and discontinuous decrease conditions were not significantly different. A possible explanation is that saliency of start and end points were diminished in our discontinuous condition compared to previous studies. In Carlile and Best^8^, the spatial configuration of the two sound stimuli had a measurable effect on performance. Rather than having overlapping trajectories for each of the distinct sounds, our stimuli were spatially adjacent. This meant that subjects could not compare the duration each sound spent traversing the common region of space, which may confer some advantage in velocity discrimination. Reduced saliency of the start and end points may also explain why the discontinuous thresholds are significantly higher than our previous study^8^.

The main finding from this experiment was that listeners were highly insensitive to instantaneous increases in velocity. This result has not been reported in any previous auditory motion studies, but has been observed for visual motion when a small aperture was used^16^. The discrepancy between increase and decrease thresholds only for the continuous transitions suggests that a different strategy or mechanism is used for perceiving changes in velocity that occur during motion. Of relevance might be the fact that the faster sounds require longer trajectories to be detected than slower sounds^17^. In the multi-snapshot framework, this could correspond to fewer snapshots being available to decide whether the velocity change has occurred. A problem with an interpretation based on motion detection thresholds is that it cannot account for the difference observed between the 30°/s increase thresholds and 60°/s decrease thresholds. Both had overlapping ranges of test velocities but increase condition had larger JNDs, indicating the order of presentation of the two constant velocity intervals influenced the perception of the velocity change. Snapshot-type models are too simplistic to explain this type of result as each estimate is assumed to be independent of any previous snapshots. Additionally, there is physiological evidence to suggest the history of stimulation of a moving sound changes the response of motion responsive cells in the inferior colliculus^18^.

A more plausible model for auditory motion perception is a leaky integrator^9^. In this model the integrator stores previous velocity estimates of the moving sound and updates this estimate via averaging as new velocity information is received. As such, some time will be required for new information to affect the mean percept. For comparable velocity integrator models in vision and touch, the integration window is around 1 second^19^. Detecting abrupt changes in velocity would be particularly difficult with an integrator mechanism, especially for increases in velocity as the total stimulus duration is decreased as opposed to decreases in velocity. Additionally, the discrepancy between sensitivity to increases and decreases is also consistent with the finding that humans have a slow velocity prior in motion perception^5^, which can be easily incorporated into an integrator model. In the discontinuous condition, the one-second gap would allow velocity information from the first interval to “leak” but may not completely reset the integrative process. It is conceivable that a significant change in spatial location produces an effective reset of the integrator, explaining the differences in sensitivity between our control condition and Carlile and Best^8^.

## Additional Information

**How to cite this article**: Locke, S. M. *et al*. Sensitivity to Auditory Velocity Contrast. *Sci. Rep*. **6**, 27725; doi: 10.1038/srep27725 (2016).

## Supplementary Material

Supplementary Information

Supplementary Audio S1

Supplementary Audio S2

Supplementary Audio S3

## Figures and Tables

**Table 1:** Reference and test velocities for the decrease and increase conditions.

Reference Velocity	Test Velocity(Decrease)	Test Velocity(Increase)
30°/s	5, 10, 15, 20, 25°/s	35, 40, 60, 90, 120°/s
60°/s	15, 30, 30, 40, 50°/s	70, 80, 120, 180, 240°/s
