# Supplementary material for: Sensitivity to Auditory Velocity Contrast
Source: Sci Rep. 2016 Jun 13;6:27725. doi: 10.1038/srep27725 (PMC4904411; doi:10.1038/srep27725)
Supplement: Supplementary Information [file srep27725-s1.doc]

Title: Sensitivity to Auditory Velocity Contrast: Supplementary.

Authors: Shannon M. Locke*^1,2^, Johahn Leung*^1^, Simon Carlile ^1,3^

Affiliate:

1. School of Medical Sciences, University of Sydney, NSW 2006 Australia

2. Department of Psychology, New York University, 6 Washington Place, New York, NY 10003, USA

3. Starkey Hearing Research Center, 2110 Shattuck st #408, Berkeley, CA 94704 USA

Sensitivity analysis

D-prime was calculated as per a Yes/No task based on the following equation (see ^1^)

d’ = Z(H) – Z(FA)

where Z(H) and Z(FA) = the z score of the hit rates and false alarm rates. The hit rates corresponded to the correct responses from the participants, and the false alarm rates were the error rates averaged across the catch trials. Three catch trial types were used: 1) the velocities of the two intervals (V_1_ and V_2_) were set to V_1_; 2) the velocities of V_1_ and V_2_ were set to V_2_ and 3) the direction of the velocities reversed, i.e., if the block of trials were testing sensitivity to increases in velocity (V_2_ > V_1_), then the catch trial will have a decrease, V_2_ less than V_1_. Each catch trial type was repeated 5 times for each block randomly interspersed; therefore block contained 50 target trials and 15 catch trials. Three different types of catch trials were used instead of just one to increase randomness. There were an insufficient number of catch trials for analyzing inter type differences.

Audio examples of moving stimuli

Audio examples of the velocity contrast stimuli and a standard velocity discrimination experiment. These were synthesized from the head related transfer function of one of the authors (SML), with velocities moving at V_1_ = 60°/s and V_2_ = 120°/s.

Audio Files Legends:

Audio1 = velocity contrast stimuli, discontinuous condition (1 second inter stimulus interval);

Audio2 = velocity contrast stimuli, continuous condition;

Audio3 = an example of previous discrimination experiment as in Carlile and Best 2002.

1. Macmillan, N. A. & Creelman, C. D*. Detection Theory: A User's Gui*de. (Lawrence Erlbaum Associates, 2005).
